# The SADDEN DEATH Study: Results from a Pilot Study in Non-ICU COVID-19 Spanish Patients

**DOI:** 10.3390/jcm10040825

**Published:** 2021-02-18

**Authors:** Carlos Nicolás Pérez-García, Daniel Enríquez-Vázquez, Manuel Méndez-Bailón, Carmen Olmos, Juan Carlos Gómez-Polo, Rosario Iguarán, Noemí Ramos-López, José Luis García-Klepzig, Marcos Ferrández-Escarabajal, Adrián Jerónimo, Eduardo Martínez-Gómez, Judit Font-Urgelles, Marcos Fragiel-Saavedra, Pilar Paz-Arias, Teresa Romero-Delgado, Zaira Gómez-Álvarez, Julia Playán-Escribano, Esther Jaén, Gianna Vargas, Elizabeth González, Eva Orviz, Irene Burruezo, Alberto Calvo, Ángel Nieto, Ángel Molino, Noël Lorenzo-Villalba, Emmanuel Andrès, Carlos Macaya, Isidre Vilacosta

**Affiliations:** 1 Instituto Cardiovascular, Hospital Clínico San Carlos, 28040 Madrid, Spain; daniel.enriquez.vazquez@gmail.com (D.E.-V.); carmen.olmosblanco@gmail.com (C.O.); jc.gomezpolo@gmail.com (J.C.G.-P.); draiguaran@gmail.com (R.I.); marcos_ferres@hotmail.com (M.F.-E.); adrijeronimo@gmail.com (A.J.); emartinezg1@gmail.com (E.M.-G.); tererd9@gmail.com (T.R.-D.); zairagomez91@gmail.com (Z.G.-Á.); julia.playan@gmail.com (J.P.-E.); carlos.macaya@salud.madrid.org (C.M.); i.vilacosta@gmail.com (I.V.); 2 Servicio de Medicina Interna, Hospital Clínico San Carlos, 28040 Madrid, Spain; manuelmenba@hotmail.com (M.M.-B.); noemi.rl92@gmail.com (N.R.-L.); josgar08@ucm.es (J.L.G.-K.); mfra2507@gmail.com (M.F.-S.); pilpazari23@gmail.com (P.P.-A.); estherjaen88@gmail.com (E.J.); evaorviz@gmail.com (E.O.); irene.burruezo@gmail.com (I.B.); ae.calvoelias@gmail.com (A.C.); anietosan@gmail.com (Á.N.); angelmanuel.molino@salud.madrid.org (Á.M.); 3 Servicio de Reumatología, Hospital Clínico San Carlos, 28040 Madrid, Spain; juditfu19@gmail.com; 4 Servicio de Neumología, Hospital Clínico San Carlos, 28040 Madrid, Spain; gvargas@alumni.unav.es (G.V.); elisabethmanuely@gmail.com (E.G.); 5 Service de Médecine Interne, Diabète et Maladies Métaboliques, Hôpitaux Universitaires de Strasbourg, 67091 Strasbourg, France; noellorenzo@gmail.com (N.L.-V.); emmanuel.andres@chru-strasbourg.fr (E.A.)

**Keywords:** COVID-19, unexpected death, mortality

## Abstract

**Introduction:** The worldwide pandemic, coronavirus disease 2019 (COVID-19) is a novel infection with serious clinical manifestations, including death. Our aim is to describe the first non-ICU Spanish deceased series with COVID-19, comparing specifically between unexpected and expected deaths. **Methods:** In this single-centre study, all deceased inpatients with laboratory-confirmed COVID-19 who had died from March 4 to April 16, 2020 were consecutively included. Demographic, clinical, treatment, and laboratory data, were analyzed and compared between groups. Factors associated with unexpected death were identified by multivariable logistic regression methods. **Results:** In total, 324 deceased patients were included. Median age was 82 years (IQR 76–87); 55.9% males. The most common cardiovascular risk factors were hypertension (78.4%), hyperlipidemia (57.7%), and diabetes (34.3%). Other common comorbidities were chronic kidney disease (40.1%), chronic pulmonary disease (30.3%), active cancer (13%), and immunosuppression (13%). The Confusion, BUN, Respiratory Rate, Systolic BP and age ≥65 (CURB-65) score at admission was >2 in 40.7% of patients. During hospitalization, 77.8% of patients received antivirals, 43.3% systemic corticosteroids, and 22.2% full anticoagulation. The rate of bacterial co-infection was 5.5%, and 105 (32.4%) patients had an increased level of troponin I. The median time from initiation of therapy to death was 5 days (IQR 3.0–8.0). In 45 patients (13.9%), the death was exclusively attributed to COVID-19, and in 254 patients (78.4%), both COVID-19 and the clinical status before admission contributed to death. Progressive respiratory failure was the most frequent cause of death (92.0%). Twenty-five patients (7.7%) had an unexpected death. Factors independently associated with unexpected death were male sex, chronic kidney disease, insulin-treated diabetes, and functional independence. **Conclusions:** This case series provides in-depth characterization of hospitalized non-ICU COVID-19 patients who died in Madrid. Male sex, insulin-treated diabetes, chronic kidney disease, and independency for activities of daily living are predictors of unexpected death.

## 1. Introduction

Based on data reported by the John Hopkins University Coronavirus Resource Center, severe acute respiratory syndrome coronavirus 2 (SARS-CoV-2) has caused more than two million deaths in 221 countries [1]. Spain, with more than 2,900,000 cases and 60,000 deaths as of February 2021, has one of the highest burdens of COVID-19 worldwide. Madrid has the highest rate of death per capita in Spain [2].

At the hospital, with the massive admission of patients, we are struggling with limited ICU capacity and resources. We prioritize patients who are more likely to survive over those with much lower chances of survival. Such prioritization inevitably leads to some patients dying from progressive respiratory failure in the hospital wards. While we were taking care of these patients, we realized that some died suddenly and unexpectedly, and that hypoxemia did not sufficiently explain the ultimate reason for death. This fact, together with the social and emotional impact of isolation during the first wave of COVID-19 pandemic, explains the name of our study. SADDEN is the result of the combination of “Sudden” and “Sad”, as most patients died alone and away from their families.

COVID-19-related deaths are not clearly defined in the available international reports. Most articles are related to critically ill ICU patients [3,4]. Thus, there is not much information on how patients die on general medical wards. Specifically, nothing has been published about sudden death and COVID-19. Our hypothesis was that some of the unexpected deaths could be cardiovascular in origin.

The purpose of this study was to describe the demographics, baseline comorbidities, clinical presentation, laboratory data, and other complementary test results of the first deceased series of hospitalized patients from an academic hospital in Spain. Specifically, we aimed to compare patients with unexpected and expected deaths.

## 2. Methods

### 2.1. Study Design and Participants

A retrospective study was conducted at Clínico San Carlos Hospital in Madrid. Patients with confirmed COVID-19 by positive reverse transcriptase-polymerase chain reaction (RT-PCR) of a nasopharyngeal sample who required hospital admission between 4 March 2020, and 16 April 2020 were included.

The flow of patients was: (1) from the Emergency Department (ED); most patients were discharged or admitted to hospital, and a minority died there;(2) among hospitalized patients, we distinguished four subgroups: patients with severe disease admitted to ICU, patients with severe disease, not admitted to ICU because of lack of resources, patients with severe disease and poor prognosis, who were not candidates for intensive care, and patients with disease not severe enough to warrant ICU; (3) Our study cohort was formed by those patients from the last three subgroups who died in our general medical wards. Due to their different clinical situation and treatment, patients who died in the ICU and in ED were excluded.

This study was approved by the local ethical committee (protocol code 20/342-E_COVID, 17 April 2020), and the study protocol was carried out in accordance with The Code of Ethics of the World Medical Association (Declaration of Helsinki).

### 2.2. Data Collection

A comprehensive multidisciplinary investigation of the clinical course, complementary test results and premortem data was performed. Data were retrospectively collected from electronic medical records and patient files; daily reports from nurses and doctors were carefully reviewed. Data included patient demographics, comorbidities, home medications, clinical status on admission, initial laboratory tests, initial electrocardiogram (12-lead ECG), inpatient medications, evolutionary ECG changes, transthoracic echocardiography, computed tomography angiography (CTA), detailed clinical evolution during the hospital course, emphasizing the last 24 h before death, length of stay, and the mode of death. For purposes of comparison, we distinguished two groups: Group I (patients with unexpected death), and Group II (expected death). The study was divided into three equal periods of a fortnight, as treatment and ICU resources varied along the study period. All data were recorded on a multipurpose database exclusively created for this study.

### 2.3. Definitions

Patients were considered to have confirmed infection if the initial RT-PCR test result was positive, or if it was negative but repeat testing was positive. Initial laboratory testing was defined as the first test results available, typically within 24 h of admission. All patients had chest X-ray and ECG at admission and, in most cases, during hospitalization. For those laboratory results, electrocardiographic traces, and other studies for which not all patients had values, percentages of total patients with completed tests are shown.

The CURB-65 score was used for the assessment of the severity of pneumonia at admission [5]. Cognitive impairment was assessed according to the Mini-Mental State Examination administered before admission [6]. Sepsis and septic shock were defined according to the 2016 Third International Consensus Definition for Sepsis and Septic Shock. Secondary infection was diagnosed when the patient had a positive culture from lower respiratory tract specimens or blood samples [7]. Resistant hypertension and chronic kidney disease (CKD) were defined according to the European Society of Cardiology guidelines [8]. Acute respiratory distress syndrome was diagnosed according to the Berlin definition [9]. Difficulty with activities of daily living (ADL) were divided into three categories (no, partial and total).

All investigators participating in this study were also deeply involved in the care of these patients. The actual role of COVID-19 in the patient’s death was classified into three groups according to the infection severity, comorbidity burden and life expectancy: Group A (no cancer, absence of comorbidities or decompensated underlying disease, and life expectancy ≥1 y); Group B (active cancer, decompensated comorbidities and/or life expectancy <1 y), and Group C (the underlying condition was the cause of death and the infection behaved as an epiphenomenon).

### 2.4. Definition of Unexpected Death

In this study, unexpected death was defined as a sudden, non-traumatic death caused by cardiac or unknown cause [10]. Although the term “sudden cardiac death” could be a synonym, we preferred to employ the term “unexpected death”, due to the particular scenario (with severe hypoxemia) of many of these patients, and the limitations on performing autopsies, especially during the first wave of the pandemic.

The death of these patients (Group I) contrasts with Group II, patients who die presenting progressive respiratory failure, often accompanied by medical sedation; ischemic or hemorrhagic stroke; sepsis; progressive heart failure; coagulopathy or multiorgan failure.

In order to be considered as unexpected death, the criteria used were as follows: in unwitnessed cases, the person had to have been seen as clinically stable <2 h before being found dead, and in witnessed cases, the patient had to have an acute change in his hemodynamic status and/or any cardiovascular symptom (chest pain, syncope, sudden breathlessness, etc.) with the time to death being <1 h. In doubtful cases, a multidisciplinary team made up of three cardiologists (CP, DE, IV), one internist (MM), and the researcher in charge of collecting patients’ data, made the final decision.

### 2.5. Statistical Analysis

Categorical variables are expressed as a frequency and a percentage and compared with the chi-square test and Fisher´s exact test as appropriate. Continuous variables are expressed as median and interquartile range (IQR) or mean and standard deviation (SD). Assessment of the normality and equality of variances for continuous data was performed using the Shapiro–Wilk test. Thereafter, continuous variables were compared using Student´s *t*-test, the Mann–Whitney U test or Wilcoxon test. To identify the variables associated with unexpected death, univariable and multivariable logistic regression models were used. Variables that were statistically significant in the univariable analysis (*p* < 0.05), or considered clinically relevant, were integrated in a multivariable regression model. The adjusted odds ratios (ORs) with 95% confidence intervals (CIs) for each variable were calculated.

All tests were two-sided, and differences were considered statistically significant at *p* values < 0.05. Statistical analysis was performed with Stata v14.1 (StataCorp, College Station, TX, USA).

## 3. Results

### 3.1. Patient Characteristics and Pre-Hospital Condition

Out of 1946 adult patients who were hospitalized between March 3 and April 16 2020 with COVID-19, we obtained data from 387 patients who died in hospital. After excluding 33 patients who died in the ICU, 16 in the ED, and 14 inpatients without available key information or confirmed infection, we included 324 deceased patients in the final analysis. The demographics and patients´ clinical characteristics are shown in Table 1. The median age was 82.8 years (IQR 76–87), ranging from 46 years to 105 years; 55.9 % were male. Importantly, one hundred and twenty-six patients (39.1%) were partially or totally dependent in ADL, and 49 (15%) were institutionalized. Twenty-two cases (6.8%) were nosocomial.

The global burden of cardiovascular diseases and risk factors was significant (65.4% had ≥2 main cardiovascular risk factors), with hypertension being the most common cardiovascular risk factor, followed by type-2 diabetes mellitus (T2DM), in 34.3%. Ninety-eight patients (30.3%) had chronic pulmonary disease (CPD) and 22 (6.8%) of them received home supplemental oxygen. One hundred and thirty patients (40.1%) had CKD, with nine in hemodialysis. Eighty-one patients (25%) were on anticoagulants and 176 (55%) were taking an angiotensin-converting enzyme inhibitor (ACE-i) or an angiotensin II receptor blocker (ARB) at home. Additional home medication can be found in the Supplementary Material.

### 3.2. Admission and Clinical Evolution

The median time from symptom onset to admission was 5 days (IQR 3–8). Patient´s characteristics at admission and clinical evolution are shown in Table 2. One hundred and thirty-two patients (40.7%) had a CURB-65 score ≥3 at admission. The most common symptoms on admission were fever, dyspnea, cough, and fatigue. Two hundred and twelve patients (66%) had bilateral pneumonia; other radiological findings are shown in the Supplementary Material. Thirty-eight patients (18.5%) were in atrial fibrillation. Seventeen patients (5.5%) had bacterial co-infection. Most patients had lymphocytopenia, and elevated levels of D-dimer, serum ferritin and C-reactive protein (Table 2). One hundred and five (32.4%) patients had an increased level of troponin I (>0.05 ng/mL) during hospitalization. Two hundred and fifty-two patients (77.8%) received antivirals, and 142 (43.8%) received corticosteroids. Both the use of corticosteroids and prophylactic anticoagulation were greater in the last fortnight than in the first two (50.7% vs. 7.0% and 42.3%, *p* < 0.001, and 62.0% vs. 26.3% and 46.7%, *p* 0.003, respectively). Corticosteroids were more frequently used when ferritin value was ≥1500 ng/mL (58.7% vs. 43.8%; *p* 0.04). Prophylactic anticoagulation was less frequently prescribed in patients with CKD (46.8% vs. 66.3%, *p* 0.02). Of the patients taking ACE-i or ARB at home, 68 continued taking these medications during their hospitalization. Fourteen patients had a QTc interval >450 ms, and only two had a QTc >500 ms; ventricular arrhythmias were not documented in these patients.

**Figure 1:**
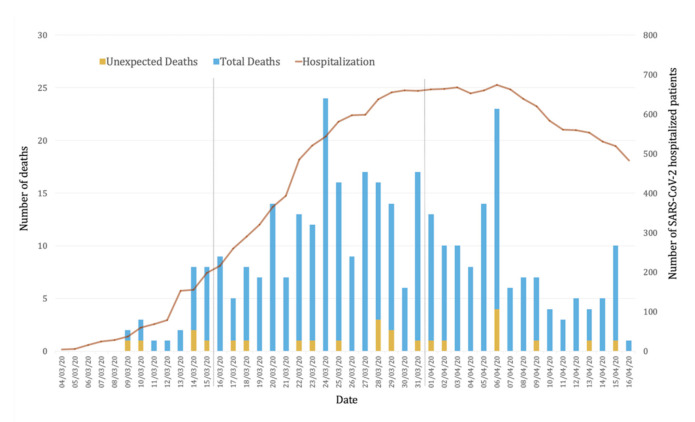
In-hospital mortality in patients with SARS-CoV-2 infection during the study period. The daily number of unexpected deaths (yellow), non-unexpected deaths (blue) and number of COVID-19 hospitalized patients are represented and divided by fortnight.

The overall length of hospital stay was 5 days (IQR 2–9). Deaths were more frequent in the second fortnight of the study (*n* = 184, 56.8%) (Figure 1). The most frequent cause of death was progressive respiratory failure (*n* = 298, 92.0%). Unexpected death occurred in 25 patients (7.7%). When we analyzed the actual contribution of the virus to the patient´s death, we found that, in 45 patients (13.9%) (Group A), the death was exclusively attributed to COVID-19, and in 254 patients (78.4%) (Group B), both COVID-19 and the patient´s clinical situation before admission contributed to death (Table 2). As expected, median age was lower in Group A than in Group B (74.9 vs. 84.2 y, *p* < 0.001). Cognitive impairment and active cancer were more common in Group B (2.2% vs. 26.1%, *p* < 0.001, and 0% vs. 54.7%, *p* 0.03, respectively). All patients from Group A were independent for ADL, whereas in Group B only 56.8% were functionally independent. There were 25 patients with COVID-19 (Group C) whose death was independent of SARS-CoV-2 infection (cardiogenic shock, multiorgan failure, aspiration pneumonia, medullary aplasia, hemorrhagic stroke, etc.).

The reason the patient was not admitted to the ICU is shown in Table 2. Increased age, high comorbidity (e.g., cirrhosis, hemodialysis, severe COPD), frailty, and cognitive impairment were the most frequent reasons. Most patients from Group B were not candidates for mechanical ventilation. Any patient over 70 years old was a borderline candidate for intensive care treatment. However, the age threshold for being an ICU candidate varied throughout the study period depending on resources, and during the period of the study, 18% of our patients were pronated on the general ward.

Among patients from Group A, the main reason for not being admitted to the ICU was the lack of ICU beds in 67.4% of cases.

### 3.3. Unexpected Death

Patients with unexpected death had a median age of 82.1 years (72.2–87.3), and 21 were male (84%). The comparison of clinical characteristics, treatment and complications between patient groups are shown in Table 3. The proportion of unexpected deaths in relation to those with expected death was higher in the first study period (26.3% vs. 6.0% vs. 7.4%, *p* 0.01). Patients with unexpected death were more frequently independent in ADL than those with expected death, whereas cognitive impairment and confusion were more common in the latter. Three patients collapsed in the bathroom and were discovered by the nurse later.

Cardiovascular risk factors, especially insulin-treated diabetes, were more frequently present in patients with unexpected death. CKD and CPD were also more frequent in these patients. Levels of D-dimer, troponin I, serum ferritin, and IL-6 were similar in both groups. There were no differences in ECG abnormalities between groups during hospitalization; nonetheless, two patients with unexpected death had an acute coronary syndrome (ACS). No patient with a long QTc interval died of unexpected death. Antiviral and corticosteroid therapy were similar between both groups.

**Figure 2:**
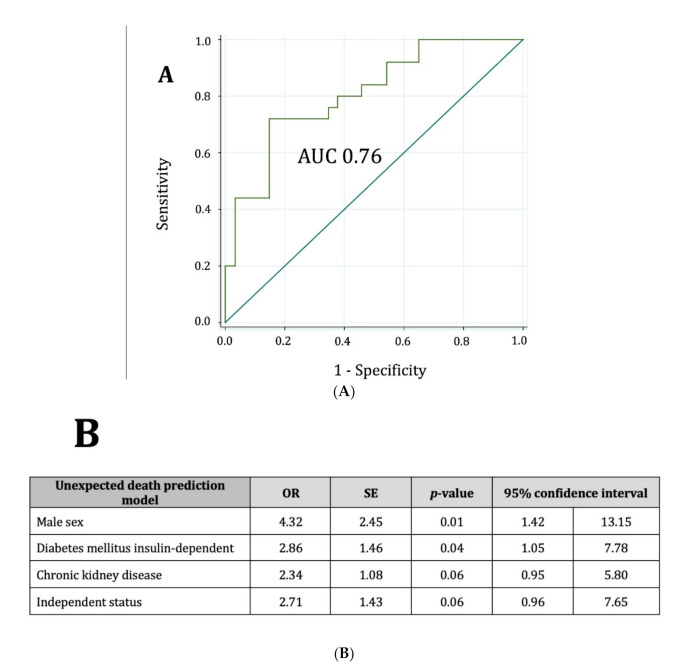
Unexpected death prediction model. (**A**) Represents the ROC curve with an area under the curve (AUC) of 0.75. Below, (**B**) Represents in a table the multivariable analysis with the corresponding values of odds ratio for each one of the four variables included in the model and their respective confidence intervals and *p*-values. OR: odds ratio, SE: standard error.

Factors significantly associated with unexpected death by univariable analysis are shown in Table 3. The variables included in the multivariable analysis were mainly those that were statistically significant in the univariable analysis (as shown in the Supplementary Material). Only male sex, insulin-treated diabetes, CKD, and independent status for ADL were independently associated with unexpected death (Figure 1). The discriminatory value of the model (AUC 0.76 (95% CI: 0.67–0.85)) is shown in Figure 2. A comparison of observed and predicted unexpected death in our cohort is shown in Supplementary Figure S1.

## 4. Discussion

This hitherto largest series of consecutive non-ICU patients with COVID-19 who died in Spain illustrates that, as in the case of Italy [3], our patient population is older (median age 82.8 y) than the average age reported in Chinese patients [4,11,12]. Mortality increases with older age and this may partly explain differences in case-fatality rates among countries. The presence of pre-existing comorbidities was also particularly important in our series. Case fatality rate is much higher in patients with comorbidities [3,4,12,13,14,15]. In the current series, cardiovascular burden was also very prevalent (35.8%), and main cardiovascular risk factors were frequently present, especially hypertension (78.4%). A higher prevalence of hypertension in patients with poor outcome has also been reported [15], but when this association is adjusted for other risk factors, there is no strong evidence to indicate that hypertension is a predictor of mortality. Surprisingly, active smoking was very uncommon in our series (*n* = 15). In other COVID-19 series, it has also been lower than expected in a population with primary respiratory infection [16]. The percentage of obese patients (18.5%) was much lower than in the series of the NY city area (41.7%) [13].

One of the most relevant aspects is the high frailty of our patient´s cohort. Almost 40% were partially or totally dependent in ADL and 15% were institutionalized. These data are, by extension, a reflection of what has happened in Madrid´s region, as 30% of the deceased were institutionalized [17].

CURB-65 at admission, a clinical predictor score validated for predicting mortality in community-acquired pneumonia, was not accurate in our cohort (60% had a score ≤2). The absence of tachypnea in many hypoxemic patients (16.4% and 40% with O_2_ saturation <90% and ≤92% on room air, respectively), what could be called silent hypoxemia, and a stable hemodynamic situation at admission, might partially explain the low CURB-65 scores. Thus, compromised respiratory status at admission (the primary driver of pneumonia severity) was not the rule. To maintain hospital safety, imaging studies were not frequently performed. Thus, we do not have enough CT studies to completely depict these patient’s pulmonary syndrome.

Corticosteroids and prophylactic heparin were more frequently used in the later, rather than in the early phase of the study, denoting that, as we became more knowledgeable about the characteristics of the disease (cytokine release storm and increased risk of venous thromboembolism), treatment changed.

In our series, 18% of patients could not benefit from ICU care due to lack of resources, and the same proportion of patients was pronated on the general ward. Similarly, a quarter of patients who died early in the Wuhan outbreak did not receive mechanical ventilation [18]. This clearly reflects the limitations of our healthcare systems, which were abruptly overwhelmed by a huge surge of patients needing mechanical ventilation. When resources are finite, differentiating whether the cause of the clinical situation is the viral infection or the patient´s underlying condition is of paramount importance. Seventy-eight percent of our cohort were not ICU candidates due to their primary clinical situation. Regardless, ICU care for the elderly is not exactly a lifeline since, in some series with COVID-19, the mortality rate in patients over 65 years who required mechanical ventilation was 97% [12,13].

It is essential to assess the actual role of COVID-19 in patient’s death. In 7.7% of our cohort (Group C), the actual contribution of the virus to the patient’s death was minimal, if any. In patients with terminal cancer, very advanced heart failure, multiorgan failure, etc., was the patient´s underlying condition the cause of death, not the viral infection.

### Unexpected Death

The actual cause of death is important, as the real course of the disease is not well known. Remarkably, 7.7% of our cohort had an unexpected death. To the best of our knowledge, there are no recorded COVID-19 cases with such unexpected deaths.

All these patients had severe pneumonia and were more frequently male. Insulin-treated diabetes, CKD and independency for ADL were independently associated with unexpected death. Several pathophysiological mechanisms (e.g., myocardial inflammation, coronary ischemia, arrhythmias, electrolyte imbalance, pulmonary embolism) can trigger these patients’ death.

Cardiac arrest occurs in about 3% of patients admitted with community-acquired pneumonia [19]. The most frequent risk factors of cardiac events in inpatients with pneumonia include older age, main cardiovascular risk factors, and greater severity of pneumonia at presentation [20]. All of them were present in our patient population. Cardiovascular risk factors, especially diabetes, were highly prevalent in patients with unexpected death. A recent meta-analysis showed a 75% increase in the relative risk of sudden death among diabetics compared to patients without diabetes. Pathogenesis involves macrovascular (coronary atherosclerosis), microvascular disease, and autonomic neuropathy [21].

Coronary artery disease, arrhythmias and sudden cardiac death represent the main causes of morbidity and mortality in patients with CKD. Pathogenesis involves the presence of traditional and non-traditional cardiovascular risk factors, including metabolic abnormalities [22]. Additionally, in our cohort, patients with CKD received prophylactic anticoagulation less frequently than those with normal renal function.

Though COVID-19 could be regarded as a disease that is confined to the lungs, cardiac complications, including myocardial infarction, myocarditis, conduction block and cardiac arrhythmias, have been reported [11,16,23]. Two patients with unexpected death had an ACS pointing to coronary artery disease (CAD) as the probable cause of death. However, increased troponin values did not significantly differ between unexpected and non-unexpected deaths. High levels of troponins have been associated with severe COVID-19 pneumonia and death [12,24]. In most patients, the reported association could be more a consequence of the systemic inflammatory response than a sign of obstructive CAD. Antivirals used during the treatment of COVID-19 may increase the risk for arrhythmias and sudden death [25]. In this series, we did not find any differences between groups regarding treatment with these drugs or QTc prolongation.

Coagulopathy (increased D-dimer concentrations and prolongation of prothrombin and activated partial thromboplastin times) was frequently present, and, although not statistically significant in the multivariable analysis, it was more common in patients with unexpected death. Coagulopathy and pulmonary embolism (PE) are highly prevalent in COVID-19 [26,27,28]. Several studies focused on autopsies have confirmed that SARS-CoV-2 patients have a baseline hypercoagulable state and are at increased risk for pulmonary thrombotic microangiopathy as well as the development of deep vein thromboses and major pulmonary thromboembolism. The autopsy findings support evaluation and management for coagulopathy early in the course of disease and judicious use of prophylactic anticoagulants while hospitalized [27,29]. In a necropsy series of 11 patients in whom PE was not clinically suspected, thrombosis of small and mid-sized pulmonary arteries was found in all patients. Ten of them had received prophylactic anticoagulation [28].

Unexpected deaths were more frequent in the first study period along with less prophylactic anticoagulation. These data underpin PE as another potential cause of unexpected death.

We do not have an adequate explanation as to why independency for ADL was related to unexpected death. The prevalence of functional disability and its association with outcomes among patients with COVID-19 has not been studied. As in heart failure, COVID-19 patients with ADL difficulty might be more prone to die from underlying diseases and progressive respiratory failure than from cardiovascular causes. On the contrary, in independent subjects, a cardiovascular origin of death could be more likely [30]. Moreover, independent patients have more personal autonomy and require less hospital supervision, what would justify why three of them were found collapsed in the bathroom. There is little doubt that cardiac arrest in the bathroom has, in most cases, a cardiovascular cause [31].

Numerous uncertainties remain in our understanding of the management of COVID-19 and on the causes of death, particularly sudden death. Improving understanding of the mechanism of death is critical. Meanwhile, we advocate for regular monitoring of independent diabetic males with CKD and severe COVID-19 pneumonia. Prophylactic anticoagulation is a must for all these patients. From a research perspective, the issues discussed highlight the need for autopsy reports to better understand this deadly disease.

## 5. Conclusions

This study provides a comprehensive overview of hospitalized non-ICU deceased patients with confirmed COVID-19 in the city of Madrid. The contribution of the virus infection to the patients’ deaths has been assessed in detail. Some patients died unexpectedly and predictors of this type of death include male sex, insulin-treated diabetes, chronic kidney disease, and independence in activities of daily living. These results suggest that unexpected death in COVID-19 might be due to cardiovascular causes.

### Limitations

This study has several limitations. Firstly, the study population only included non-ICU hospitalized deceased patients, so interpretation of our findings is limited to this patient population. Secondly, due to the retrospective study design, not all tests were recruited in all patients. Therefore, their role, especially the 48 h ECG after admission, might be underestimated in predicting unexpected death. Moreover, as patients were admitted to general medical wards, continuous monitoring was not available.

## Figures and Tables

**Table 1:** Baseline characteristics of study population.

Baseline Characteristics	Total of Deaths (*n* = 324)
**Demographic characteristics**	
Age (median (IQR))—y. Age (years)	82.8 (76.5–87.3)
Male gender—no. (%)	181 (55.9)
**Frailty indicators**	
Functional dependency—no. (%) *n* = 322	
Independent	196 (60.9)
Partially dependent	87 (27)
Totally dependent	39 (12.1)
Institutionalization—no. (%)	49 (15.1)
Cognitive impairment—no. (%)	74 (22.8)
**Cardiovascular risk factors**	
Hypertension—no. (%)	254 (78.4)
Resistant hypertension—no. (%)	38 (11.7)
T2DM—no. (%)	111 (34.3)
T2DM–OAD	90 (27.8)
T2DM-insulin	47 (14.5)
Hyperlipidemia—no. (%)	187 (57.7)
Obesity—no. (%)	60 (18.5)
Current smoking—no. (%)	15 (4.6)
Former smoking—no. (%)	95 (30.9)
<2 CVRF—no. (%)	112 (34.6)
≥2 CVRF—no. (%)	212 (65.4)
**Cardiovascular history**	
Ischaemic heart disease—no. (%)	40 (12.3)
Prior MI—no. (%)	28 (8.6)
Prior stroke/TIA—no. (%)	37 (11.4)
Atrial fibrillation—no. (%)	62 (19.1)
Pacemaker—no. (%)	19 (5.9)
ICD ± CRT—no. (%)	2 (0.6)
LVEF < 35 % *n* = 198	8 (4.0)
**Other comorbidities**	
Chronic pulmonary disease—no. (%)	98 (30.3)
COPD	49 (15.1)
Asthma	14 (4.3)
OSAS	16 (4.9)
Restrictive disorder	15 (4.6)
Chronic kidney disease—no. (%)	130 (40.1)
Chronic liver disease—no. (%)	17 (5.2)
Rheumatological disease—no. (%)	31 (9.6)
Hypothyroidism—no. (%)	38 (11.7)
Active cancer—no. (%)	42 (13.0)
Immunosuppression *—no. (%)	55 (17.0)

Values are no (%) or median (interquartile range) as indicated. * Combination of immunosuppressive therapies (corticosteroids and other immunosuppressive drugs), transplanted patients and HIV+. COPD: chronic obstructive pulmonary disease, CRT: cardiac resynchronization therapy, CVRF: cardiovascular risk factor, HIV: human immunodeficiency virus, ICD: implantable cardioverter defibrillator; MI: myocardial infarction, no: number, OSAS: obstructive sleep apnea syndrome, T2DM: type-2 diabetes mellitus.

**Table 2:** Patient characteristics during admission.

Patient Characteristics during Hospitalization	Total of Deaths (*n* = 324)			
**Clinical presentation**				
Fever ≥38 °C—no. (%)	214 (66.3)			
Cough—no. (%)	191 (59.1)			
Dyspnea—no. (%)	204 (63.0)			
Asthenia—no. (%)	139 (43.0)			
Anosmia—no. (%)	3 (0.9)			
Ageusia—no. (%)	6 (1.9)			
Gastrointestinal symptoms—no. (%)	91 (28.1)			
Syncope—no. (%)	12 (3.7)			
Myalgia—no. (%)	42 (13.0)			
Confusion—no. (%)	91 (28.4)			
Time from symptoms to admission (median (IQR))–days.	5 (3–8)			
Clinical situation at admission				
CURB-65 >2—no. (%)	132 (40.7)			
pH (venous sample) (mean ± SD)	7.4 ± 0.1			
pCO2 (venous sample, mmHg) (mean ± SD)	40.7 ± 9.9			
Capillary oxygen saturation (%) (mean ±SD)	87.2 ± 9.1			
Systolic blood pressure (mmHg) (mean ± SD)	127.8 ± 23.9			
Diastolic blood pressure (mmHg) (mean ± SD)	70.2 ± 13.9			
Tachypnea—no. (%)	150 (47.9)			
Abnormal pulmonary auscultation (rales, hypophonesis and rhonchus)—no. (%)	296 (91.4)			
Time from symptoms to initiation of therapy (median (IQR))—days.	5 (2–7)			
Clinical evolution (24 h before death)				
Acute respiratory and radiological worsening—no. (%)	299 (92.9)			
Pronation in bed (awake)—no. (%)	58 (18.0)			
Hemodynamic instability—no. (%)	115 (36.5)			
New onset arrhythmias—no. (%)	8 (2.6)			
Neurological deterioration—no. (%)	100 (32.8)			
Acute renal injury—no. (%)	94 (30.2)			
Chest pain—no. (%)	7 (2.2)			
Syncope—no. (%)	4 (1.2)			
Length of hospitalization (median (IQR))—days.	5 (2–9)			
Time from initiation of therapy to death (median (IQR))—days.	5 (3–8)			
Laboratory findings				
Hemoglobin at admission (mean ± SD)—g/dL	13.0 ± 2.1			
Leucocytes at admission (mean ± SD)—cells/mm^3^	8757.8 ± 7427.4			
Platelets at admission (mean ± SD)—cells/mm^3^	185,467 ± 90,082			
CRP at admission (mean ± SD)—mg/dL	13.9 ± 10.4			
Procalcitonin at admission [median (IQR)]—ng/mL	0.3 (0.1–0.7)			
Creatinine at admission (mean ± SD)—mg/dL	1.6 ± 1.6			
Urea at admission (mean ± SD)–mg/dL	85.2 ± 87.0			
Maximum lymphocytopenia *—(median (IQR))—cells/mm^3^	500 (300–700)			
Peak Troponin I † (mean ± SD)–ng/mL	2.4 ± 26.1			
Peak D-dimer (mean ± SD)–ng/mL	10,808 ± 25646			
Peak Ferritin (mean ± SD)–ng/mL	1385 ± 1673			
Peak Triglycerides (peak) (mean ± SD)–mg/dL	179.9 ± 138.0			
IL-6 (median (IQR))—ng/mL	86.3 (51.5–273)			
INR (24 h before death) (mean ± SD)	1.7 ± 2.1			
APTT (24 h before death) (mean ± SD)—s	30.7 ± 9.4			
ECG findings				
Sinus rhythm–no. (%)	162 (79.4)			
QT interval corrected ‡ (mean ± SD)—ms.	403.0 ± 38.6			
PR interval (mean ± SD)—ms.	191.4 ± 35.7			
**Treatment during hospitalization**				
Hydroxychloroquine–no. (%)	215 (66.6)			
Lopinavir/ritonavir–no. (%)	111 (34.3)			
Darunavir/cobicistat–no. (%)	23 (7.1)			
Azithromycin–no. (%)	93 (28.7)			
Corticosteroids–no. (%)	142 (43.8)			
Tocilizumab–no. (%)	15 (4.6)			
Therapeutic anticoagulation (AVK, heparin and DOAC)–no. (%)	72 (22.2)			
Therapeutic anticoagulation per fortnight—no. (%)	**1st** 4 (21.1)	**2nd** 40 (21.7)	**3rd** 28 (23.1)	*p* 0.95
Prophylactic anticoagulation per fortnight—no. (%)	**1st** 5 (26.3)	**2nd** 86 (46.7)	**3rd** 75 (62.0)	*p* 0.003
**COVID-19 death characteristics**				
Death mainly from COVID-19 (Group A)—no. (%)	45 (13.9)			
Death from COVID-19 with comorbidity (Group B)—no. (%)	254 (78.4)			
Death with COVID-19 (Group C) §—no. (%)	25 (7.7)			
Progressive respiratory failure to death—no. (%)	298 (92.0)			
Unexpected death—no. (%)	25 (7.7)			
Death at night (22–8 h)—no. (%)	116 (41.6)			
**Reasons for non-admission to the ICU**				
Age—no. (%)	240 (74.5)			
High comorbidity—no. (%)	240 (74.5)			
Cancer—no. (%)	52 (16.0)			
Frailty—no. (%)	185 (57.1)			
Cognitive impairment—no. (%)	74 (22.8)			
Non-available ICU bed—no. (%)	59 (18.2)			
Non-available ICU bed per fortnight—no. (%) N = 59	**1st** 6 (10.1)	**2nd** 32 (54.2)	**3rd** 21 (35.6)	*p* 0.33
ICU team assessment—no. (%)	48 (14.8)			

Values are n (%), mean (± standard deviation) or median (interquartile range) as indicated. * Five patients were excluded (3 with chronic lymphocytic leukemia and 2 with lymphomas). † A value > 0.05 ng/mL was considered abnormal. ‡ Bazett formula. § Short life-expectancy before the infection. APTT: activated partial thromboplastin time, AVK: antivitamin K, CRP: C-reactive protein, DOAC: direct oral anticoagulant, ICU: Intensive Care Unit, INR: international normalized ratio, no: number, pCO2: partial pressure of carbon dioxide.

**Table 3:** Univariable analysis between patients with unexpected death and non-unexpected death.

Univariable Analysis	Unexpected Deaths (*n* = 25)	Non-Unexpected Deaths (*n* = 299)	*p*-Value
**Demographic characteristics**			
Age (median, (IQR))—y.	82.1 (72.2–87.3)	82.8 (76.8–87.5)	1.00
Male gender—no. (%)	21 (84.0)	160 (53.5)	**0.003**
**Frailty indicators**			
Functional independence—no. (%)	20 (80.0)	176 (59.3)	**0.04**
Cognitive impairment—no. (%)	2 (8.0)	72 (24.2)	0.08
**Cardiovascular risk factors**			
Hypertension—no. (%)	21 (84.0)	233 (77.9)	0.62
Diabetes mellitus 2—no. (%)	14 (56.0)	97 (32.4)	**0.02**
T2DM–OAD	7 (28.0)	83 (27.8)	0.98
T2DM–insulin requiring	8 (32.0)	39 (13.0)	**0.01**
Former smoking—no. (%)	12 (48.0)	83 (29.3)	**0.05**
2 CVRF—no. (%)	21 (84.0)	191 (63.9)	**0.049**
**Cardiovascular events**			
Prior MI—no. (%)	1 (4.0)	27 (9.1)	0.71
Prior stroke/TIA—no. (%)	2 (8.0)	35 (11.7)	0.75
Heart failure *—no. (%)	5 (21.7)	55 (19.3)	0.79
Venous thromboembolic disease (PE or DVT)	1 (4.0)	23 (7.7)	1.00
**Other comorbidities**			
Chronic pulmonary disease—no. (%)	12 (48.0)	86 (28.8)	**0.04**
Chronic kidney disease—no. (%)	16 (64.0)	114 (38.13)	**0.01**
Chronic liver disease–no. (%)	2 (8.0)	15 (5.0)	0.63
Active cancer—no. (%)	4 (16.0)	38 (12.7)	0.55
Immunosuppression †—no. (%)	7 (28.0)	48 (16.1)	0.13
**Baseline medications**			
RAAS inhibitors ‡—no. (%)	13 (52.0)	161 (53.9)	0.86
Antibiotics with effects on QT—no. (%)	3 (12.0)	35 (11.7)	1.00
Antipsychotic drugs—no. (%)	0 (0.0)	34 (11.4)	0.09
**Clinical presentation**			
Fever—no. (%)	16 (64.0)	198 (66.4)	0.80
Oxygen saturation (mean, (SD))	87.1 (±9.2)	87.2 (±9.1)	0.97
Tachypnea—no. (%)	12 (50.0)	138 (47.8)	0.83
Confusion—no. (%)	5 (20.0)	86 (29.2)	**0.05**
Abnormal pulmonary auscultation—no. (%)	20 (80.0)	276 (92.3)	**0.05**
**CURB-65 > 2—no. (%)**	**10 (40.0)**	**122 (40.8)**	**0.94**
Time from symptoms to treatment (median (IQR))—days.	4.5 (2.8–8.5)	4 (2–7)	0.85
**Clinical evolution (24 h before death)**			
Acute respiratory and radiological worsening—no. (%)	18 (72.0)	280 (94.3)	**<0.001**
Hemodynamic instability—no. (%)	7 (29.2)	108 (37.1)	0.44
New onset arrhythmias—no. (%)	0 (0.0)	8 (2.8)	1.00
Neurological deterioration—no. (%)	3 (12.0)	97 (34.6)	**0.03**
Acute kidney injury—no. (%)	6 (24.0)	88 (31.0)	0.48
**Laboratory findings**			
Creatinine at admission (mean (SD))—mg/dL	2.3 (±2.6)	1.6 (±1.4)	**0.04**
Maximum lymphocytopenia (median (IQR))—cells/mm3	500 (300–675)	500 (300–700)	0.50
Peak CRP (mean (SD))—mg/dL	15.5 (±7.9)	13.7 (±10.5)	0.42
Peak TnI (mean (SD))—ng/mL	0.26 (±0.5)	2.6 (±27.3)	0.73
Peak TnI > 0.05 ng/mL—no. (%)	9 (56.3)	96 (51.6)	0.72
Peak D-dimer (mean (SD))—ng/mL	12,207 (±28,511)	13,303 (±44,347)	0.93
Peak ferritin (mean (SD))—ng/mL	1.698 (±2030)	1,359 (±1642)	0.40
Peak triglycerides (mean (SD))—mg/dL	139 (±148.6)	327 (±726)	0.67
IL-6 (median (IQR))—ng/mL	54.3 (53.7–182.7)	103.2 (47.7–253.7)	1.00
INR (24 h before death) § (mean (SD))	2.3 (±4.5)	1.3 (±0.7)	**0.004**
APTT (24 h before death) (mean (SD))—s	35.3 (±16.4)	30.2 (±8.4)	**0.03**
**Radiological findings**			
Bilateral pneumonia	14 (56.0)	198 (66.7)	0.52
**ECG abnormalities**			
QTc (mean (SD))—ms	409 (±21)	403 (±40)	0.64
PR (mean (SD))—ms	183 (±29)	192 (±36)	0.44
**Treatments during the hospitalization**			
Hydroxychloroquine—no. (%)	17 (68.0)	198 (66.0)	0.87
HCQ + Azithromycin—no. (%)	4 (16.0)	62 (20.7)	0.80
HCQ + Lopinavir/ritonavir+ Azithromycin—no. (%)	2 (8.0)	28 (9.4)	1.00
Corticosteroids—no. (%)	10 (40)	131 (43.8)	0.89
Tocilizumab—no. (%)	1 (4.0)	14 (4.7)	1.00
Therapeutic anticoagulation—no. (%)	7 (28.0)	65 (21.7)	0.47
Prophylactic anticoagulation—no. (%)	13 (52.0)	152 (51.2)	0.94
Time from treatment to death (median (IQR))—days.	4 (3–9)	5 (2–7.8)	0.18

Values are n (%), median (interquartile range) or mean (standard deviation) as indicated. * Previous admissions for heart failure. † Combination of immunosuppressive therapies (corticosteroids and other immunosuppressive drugs), transplanted patients and HIV+. ‡ At least one of: ACE-I, ARB, MRA, or sacubitril/valsartan. § Patients in treatment with AVK or DOAC during hospitalization were excluded. CRP: C-reactive protein, CVRF: cardiovascular risk factor, DVT: deep venous thrombosis, HCQ: hydroxychloroquine, MI: myocardial infarction, no: number, OAD: oral antidiabetic; PE: pulmonary embolism, RAAS: renin–angiotensin–aldosterone system, TIA: transient ischaemic attack, T2DM: type-2 diabetes mellitus, TnI: troponin I.

## Data Availability

The data presented in this study are available on request from the corresponding author.

## Supplementary Materials

Supplementary materials can be found at https://www.mdpi.com/2077-0383/10/4/825/s1. Supplementary Figure S1. Observed versus predicted unexpected death. Observed unexpected deaths are represented by blue bars and predicted unexpected deaths by yellow bars, divided in quintiles. Supplementary Table S1. Baseline medications. Supplementary Table S2. Radiological findings.

## Abbreviations and Acronyms

ACE-i	angiotensin-converting enzyme inhibitor
ACS	acute coronary syndrome
ADL	activities of daily living
APTT	activated partial thromboplastin time
ARB	angiotensin II receptor blocker
AUC	area under the curve
AVK	antivitamin K
BP	blood pressure
BUN	blood urea nitrogen
CAD	coronary artery disease
CI	confidence interval
CKD	chronic kidney disease
COPD	chronic obstructive pulmonary disease
CPD	chronic pulmonary disease
CRP	C-reactive protein
CRT	cardiac resynchronization therapy
CTA	computed tomography angiography
CVRF	cardiovascular risk factor
CURB-65	confusion, BUN, respiratory rate, systolic blood pressure, age ≥ 65
DOAC	direct oral anticoagulant
DVT	deep venous thrombosis
ECG	electrocardiogram
ED	emergency department
HCQ	hydroxychloroquine
ICD	implantable cardiac device
ICU	intensive care unit
INR	international normalized ratio
IQR	interquartile range
LVEF	left ventricular ejection fraction
MI	myocardial infarction
MRA	mineralocorticoid receptor antagonist
no	number
OAD	oral antidiabetic
OSAS	obstructive sleep apnea syndrome
OR	odds ratio
PE	pulmonary embolism
RAAS	renin-angiotensin-aldosterone system
ROC	receiver operating characteristic
RT-PCR	reverse transcriptase-polymerase chain reaction
SARS-CoV-2	severe acute respiratory syndrome coronavirus 2
SD	standard deviation
SE	standard error
TIA	transient ischaemic attack
TnI	troponin I
T2DM	type-2 diabetes mellitus
SARS-CoV-2	severe acute respiratory syndrome-coronavirus 2
