# Supplementary material for: The SADDEN DEATH Study: Results from a Pilot Study in Non-ICU COVID-19 Spanish Patients
Source: J Clin Med. 2021 Feb 18;10(4):825. doi: 10.3390/jcm10040825 (PMC7922313; doi:10.3390/jcm10040825)
Supplement: Supplementary file 1 [file jcm-10-00825-s001.pdf]

Supplementary material

Supplementary Table 1. Baseline medications.

Baseline medications.	Total of deaths (n = 324)
**Cardiovascular treatments**—no. (%)	
ASA	89 (27.5)
AVK	57 (17.6)
DOAC	24 (7.4)
Beta-blockers	88 (27.2)
ACE-i	106 (32.7)
ARB	70 (21.6)
MRA	21 (6.5)
Sacubitril/valsartan	3 (0.9)
Statins	137 (42.2)
Calcium-channel blockers	73 (22.5)
Diuretics	119 (36.7)
Amiodarone	6 (1.9)
Other antiarrhythmic drugs	4 (1.2)
**Immunosuppressive therapies**—no. (%)	
Corticosteroids	40 (12.35)
Other immunosuppressive drugs	29 (8.9)
**Antibiotics**—no. (%)	
Potential QT prolongation	38 (11.7)
Without effects on QT	23 (7.1)
**Antipsychotic drugs**—no. (%)	
1st generation	2 (0.6)
2nd generation	26 (8.0)
3rd generation	2 (0.6)
Combinations	4 (1.2)
**Antidepressant drugs**—no. (%)	
SSRI	28 (8.6)
SNRI	8 (2.5)
TCAs TCA	1 (0.3)
Others	21 (6.5)
Combinations	15 (4.6)

Values are n (%) as indicated. ACE-i: angiotensin converting enzyme-inhibitor: ARB: angiotensin-II receptor blocker, ASA: acetylsalicylic acid, AVK: antivitamin K, DOAC: direct oral anticoagulant, MRA: mineralocorticoid receptor antagonist, SNRI: serotonin and norepinephrine reuptake inhibitor, SSRI: selective serotonin reuptake inhibitor, TCA: tricyclic antidepressant.

Supplementary Table 2. Radiological findings.

Radiological findings	Total of deaths (n = 324)
Lobar pneumonia—no. (%)	39 (12.0)
Multi-lobar pneumonia—no. (%)	63 (19.4)
Bilateral pneumonia—no. (%)	213 (66.2)
Pleural effusion—no. (%)	34 (10.5)
Non-specific radiological findings—no. (%)	37 (11.4)

Values are n (%) as indicated. Values are n (%). ACE-i: angiotensin converting enzyme-inhibitor: ARB: angiotensin-II receptor blocker, ASA: acetylsalicylic acid, AVK: antivitamin K, DOAC: direct oral anticoagulant, MRA: mineralocorticoid receptor antagonist, SNRI: serotonin and norepinephrine reuptake inhibitor, SSRI: selective serotonin reuptake inhibitor, TCA: tricyclic antidepressant.
